# Dementia and functional decline in patients with Parkinson’s disease

**DOI:** 10.1590/S1980-57642009DN20200004

**Published:** 2008

**Authors:** Florindo Stella, Claudio Eduardo Muller Banzato, Elizabeth Maria Aparecida Barasnevicius Quagliato, Maura Aparecida Viana, Gustavo Christofoletti

**Affiliations:** 1 Biosciences Institute, Campus of Rio Claro, Unesp – Sao Paulo State University , Brazil and Geriatric Psychiatry Clinic of State University of Campinas (Unicamp), Brazil; 2 Department of Psychiatry, Medical School, State University of Campinas (Unicamp), Brazil; 3 Department of Neurology, Movements Disorders Clinic, State University of Campinas (Unicamp), Brazil; 4 PhD Student on Neurosciences, State University of Campinas (Unicamp), Brazil.

**Keywords:** Parkinson’s disease, functional decline, dementia

## Abstract

Functional decline in Parkinson’s disease (PD), characterized by reduced ability to carry out activities of daily living, usually results from typical motor impairment and may be aggravated by concomitant cognitive impairment.

**Objective:**

To compare the functional decline in Parkinson’s disease between patients with dementia and cognitively preserved patients.

**Methods:**

From an original sample composed of 50 patients with a clinical diagnosis of idiopathic PD seen in a consecutive series, 33 non-depressed patients were selected comprising 13 with dementia and 20 cognitively preserved individuals. All patients enrolled in this study were drawn from a public outpatient clinic, specialized in movement disorders. The clinical stage of PD was determined by the Hoehn & Yahr scale, and the functional capacity was verified using the Unified Parkinson’s Disease Rating Scale UPDRS ADL (subscale II: activities of daily living) and the Schwab & England scale. The two last scales measure the functional degree of independence in activities of daily living. The neuropsychological assessment was performed using The Cambridge Examination for Mental Disorders of the Elderly – CAMCOG, Cognitive Section and the Stroop Color Word Test.

**Results:**

As expected, in comparison with cognitively preserved patients, the group with dementia presented significantly lower scores throughout the neuropsychological evaluation. The patients with dementia were found to have a longer period of disease, a more advanced clinical staging according to the Hoehn & Yahr, and greater functional decline according both to the UPDRS ADL and Schwab & England, with statistically significant difference between the groups.

**Conclusion:**

Patients with dementia were at a more advanced clinical stage of Parkinson’s disease and evidenced greater functional decline in comparison with patients without dementia.

## Abstract (pt)

O declínio funcional na doença de Parkinson (DP), caracterizado pela redução da capacidade para as atividades de vida diária, usualmente resulta do comprometimento motor típico, podendo ainda ser agravado pelo declínio cognitivo concomitante.

**Objetivos:**

Comparar o declínio funcional de pacientes com e sem demência na doença de Parkinson.

**Métodos:**

De uma amostra original composta por 50 pacientes com diagnóstico clínico de DP idiopática, avaliados em uma série consecutiva, foram selecionados 33 pacientes não deprimidos, sendo 13 com demência e 20 cognitivamente preservados. Todos os pacientes eram acompanhados em um ambulatório público especializado em distúrbios do movimento. Aplicamos a Unified Parkinson’s Disease Rating Scale – UPDRS (sub-escala II: atividades de vida diária – UPDRS ADL), a escala Schwab & England, a escala Hoehn & Yahr, o Cambridge Examination for Mental Disorders of the Elderly – Seção Cognitiva (CAMCOG) e o Stroop Color Word Test.

**Resultados:**

Como esperado, em comparação com pacientes cognitivamente preservados, o grupo com demência apresentou escores menores na avaliação neuropsicológica. Os pacientes com demência tinham, também, um tempo maior de doença, pertenciam a um estágio clínico mais avançado segundo a Hoehn & Yahr, e seu declínio funcional, segundo a UPDRS ADL e a Schwab & England, mostrava-se mais acentuado, com diferença estatisticamente significativa entre os grupos.

**Conclusão:**

Os pacientes com demência encontravam-se em um estágio clínico mais avançado da doença e seu declínio funcional mostrou-se mais acentuado em comparação com os pacientes sem demência.

Functional decline, a phenomenon expected in Parkinson’s disease (PD), is characterized by a reduced ability to carry out activities of daily living.^1^ Typically resulting from motor dysfunction, the difficulty in performing these activities also depends on cognitive status, especially the executive processes.^2^

Functional decline has been associated primarily with motor impairment, but less related to cognitive decline,^1^ where this is an important condition which leads to an increase in patient suffering and family burden.^2,3^ Compared with Alzheimer’s dementia, the number of studies on functional decline in Parkinson’s dementia is significantly lower, although the difference of prevalence between these diseases should be taken into account.^4,5^

The Unified Parkinson’s Disease Rating Scale (UPDRS)^6^ is a commonly applied instrument for assessment of the clinical condition of patients with PD chiefly evaluating motor symptoms. The subscale II of UPDRS assesses activities of daily living (UPDRS ADL) and together with the Schwab & England^7^ has been used to evaluate the functionality of patients with PD.^8^

This study aimed to assess the overall functional decline in non-depressed patients diagnosed with Parkinson’s disease, and compare dementia patients with cognitively preserved individuals.

## Methods

From an original sample composed of 50 patients with a clinical diagnosis of idiopathic PD seen in a consecutive series, 33 non-depressed patients were selected, comprising 13 with dementia and 20 who were cognitively preserved. The comparison between depressed and non-depressed patients with PD is presented elsewhere. The patients enrolled in this study were drawn from a public outpatient clinic, specialized in movement disorders (HC-Unicamp, Brazil). All patients were regular users of anti-parkinsonian medication such as levodopa, dopaminergic agonists or other drugs, and all were assessed during the on-phase of medication. Only one patient took regular anti-cholinesterasics (rivastigmine).

### Procedures

The demographics were identified using an appropriate questionnaire. The inclusion criteria for diagnosis of idiopathic PD followed the recommendations of the United Kingdom Parkinson’s Disease Society operative criteria.^9^ The patients were diagnosed by the outpatient clinic team of experts. We excluded those patients with movement disorders, not compatible with the diagnosis of idiopathic PD, such as secondary Parkinsonism as well as individuals with depression. Patients without regular treatment or at the *off*-phase of medication were also excluded. The decision to include only patients at the *on*-phase was based on the possible discomfort caused by non-medication. Patients enrolled in the study were undergoing regular PD treatment for at least two years. Examination of the mental condition and a neuropsychological assessment were performed. The CAMCOG (The Cambridge Examination for Mental Disorders of the Elderly – Cognitive Section)^10,11^ and the Stroop Color Word Test^12^ were used. The patients were then divided into two groups: with and without dementia, according to the clinical criteria of the DSM-IV-TR^13^ and the criteria suggested by the Task Force from the Study of Movement Disorders.^14^ Patients with dementia presented a moderate clinical profile, according to the Clinical Dementia Rating. Data on the age at onset and the duration of PD, the onset type (tremor or rigidity), the hemibody of the first clinical manifestation and the drugs prescribed were all collected. The clinical stage of PD was determined by the Hoehn & Yahr scale,^15^ and the functional capacity was verified using the Unified Parkinson’s Disease Rating Scale UPDRS ADL (subscale II: activities of daily living).^6,7^ The two last scales measure the degree of functional independence degree activities of daily living.

### Statistical analysis

The data were analyzed primarily by descriptive statistics. The software used for analysis was the Statistical Package for Social Sciences (SPSS 10.0). The independent Student’s t test was carried out to verify possible differences regarding both groups. A two-tailed significance level of 5% was adopted for this analysis (p<0.05).

## Results

**Figure 1:**
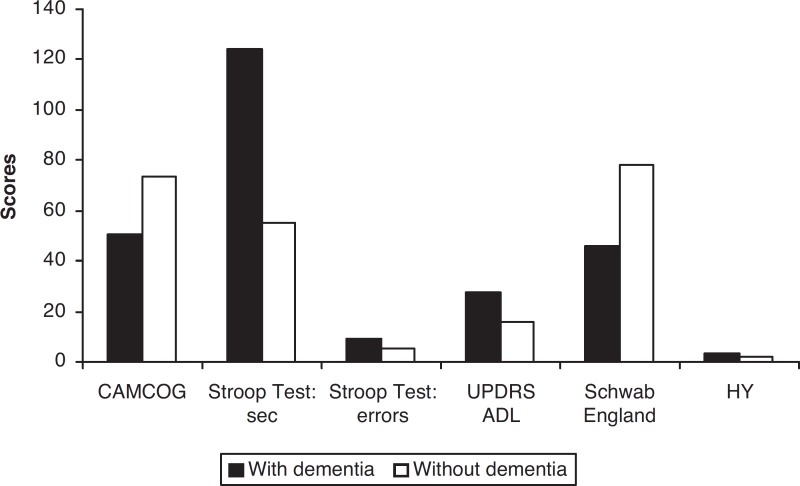
Cognitive im pairment, functional decline and Hoehn & Yahr of PD patients with and without dementia. CAMCOG: The Cambridge Examination for Mental Disorders of the Elderly (Cognitive Session); ADL: activities of daily living; HY: Hoehn & Yahr; sec: seconds; UPDRS ADL: Unified Parkinson’s Disease Rating Scale – activities of daily living.

When compared with the cognitively preserved subjects, the patients with dementia were found to have a longer disease period due to a more advanced clinical stage, according to Hoehn and Yahr. They also showed greater functional decline as measured by UPDRS ADL and Schwab & England, with significant difference between the groups. As expected, the group with dementia obtained lower scores on the CAMCOG and Stroop Test – ‘time’ category (seconds). However, for the ‘word error’ category of these tests, there was no difference between the groups. The groups were similar in terms of type of disease onset (tremor or rigidity) and hemibody at onset of clinical manifestation, except for two patients without dementia, whose first presentation was cervical region impairment (Table 1 and Figure 1).

**Table 1:** Summary of demographics and clinical features of patients with and without dementia.

Characteristics	With dementia	Without dementia	Student's t test
**Demographics**			
Patients (N=33)	13 (39.4%)	20 (60.6%)	t=1.3; p>0.05
Age (years)	70.7 (SD=7.0)	67.4 (SD=7.0)	t=0.3; p>0.05
Schooling (years)	4.4 (SD=2.9)	4.4 (SD=3.7)	
**PD clinical data**			
Age at onset of PD	57.5 (SD=8.5)	58.7 (SD=8.7)	t=0.4; p>0.05
Duration of PD	13.2 years (SD=6.0)	8.7 (SD=4.9)	t=12.3; p<0.05*
PD onset type			
- tremor 6 patients (46.1%)	8 patients (40.0%)		
- rigidity 7 patients (53.9%)	12 patients (60.0%)		
Beginning of PD in hemibody			
- right	5 patients (38.5%)	8 patients (40.0%)	
- left	8 patients (61.5%)	10 patients (50.0%)	
- cervical	---	2 patients (10.0%)	
Hoehn & Yahr	3.6 (SD=1.0)	2.2 (SD=0.8)	t=4.3; p<0.05*
UPDRS II (ADL)	27.3 (SD=11.5)	15.6 (SD=5.1)	t=3.5; p<0.05*
Schwab & England	46.1 % (SD=28.1)	78.5 (SD=12.2)	t=3.9; p<0.05*
**Neuropsychological assessment**			
CAMCOG	50.4 (SD=12.7)	73.6 (SD=7.7)	t=6.5; p<0.05*
Stroop test (seconds)	124.4 (76.5)	55.5 (SD=20.8)	t=2.9; p<0.05*
Stroop test (word errors)	9.18 (SD=9.2)	5.3 (SD=8.4)	t=1.1; p>0.05

* Significant values; PD: Parkinson's disease; UPDRS ADL: Unified Parkinson's Disease Rating Scale - activities of daily living; CAMCOG: The Cambridge Examination for Mental Disorders of the Elderly (Cognitive Session); SD: standard deviation.

## Discussion

The patient’s ability to perform his/her daily activities independently is a predicting factor for quality of life in PD, where the limitations associated with PD may be further aggravated by a cognitive impairment.^4,16^ This combination is likely to increase the family burden, which may result in early institutionalization of the patient.^2,17^

In the present study, PD patients with dementia presented a worse clinical condition, according to the Hoehn & Yahr scale, while their functional decline, according to the UPDRS ADL and Schwab & England scales, was more marked than those without dementia. These data also suggested that patients without dementia were more preserved in activities of daily living in comparison to patients with dementia. These scales have been traditionally used for investigating functional capacity in PD,^1,18^ with high internal consistency and correlation between them.^19^ Recently, these scales were used to investigate the relationship between functional decline and quality of life in PD.^16^ In a Brazilian study, they were also applied to assess functional capacity^20^ with significant positive correlation with quality of life. However, unlike our study, the above-cited studies either excluded patients with cognitive impairment or did not establish a distinction between groups with and without dementia. The correlation between functional decline and cognitive impairment observed in our work may be compared with another study that also found correlation between these variables using the same scales.^8^ Our results also may be compatible with another study that used the Functional Assessment Staging scale to measure the functional capacity of patients with PD.^4^

Although dementia in PD is classically characterized by bradyphrenia and progressive changes in executive functions, other cognitive processes are also affected, such as memory, language, orientation, praxis and recognition.^21,22^ From a neurobiological point of view, depletion of several neurotransmitter systems – dopaminergic, serotonergic, noradrenergic and cholinergic – has been associated particularly to non-motor impairment, affecting both cognitive and functional conditions.^17,21-25^ Disorders of these cognitive functions are characteristic of dementia in PD.^21,26^

Braak et al.^27^ performed a *post mortem* study of the brain of patients with PD and proposed six neuropathological progressive stages related with the severity of disease course. Several years ago, Braak et al.^23^ correlated the cognitive decline of patients, evaluated *in vivo*, with the neuropathological stages topographically verified in the autopsy procedure. These authors suggested that cognitive deficits in PD may not be attributed to isolated or confined lesions (such as Lewy bodies and deposition of alpha synuclein) but rather to progressive and cumulative amounts of these pathological phenomena. Braak et al.^23^ also reported that stages 1 and 2 are considered preclinical stages; in stage 3 there is a involvement of the *substantia nigra*; whilst stage 4 is related to involvement of other deep nuclei of the midbrain and the forebrain. Unsurprisingly, motor symptoms other non-motor symptoms have been observed. In addition, the destruction of the neocortex was verified in stages 5 and 6, interfering directly with normal cortical functions. Cortical damage of the temporal region, in particular hippocampal formation and entorhinal cortex, have been associated with memory disorders; and degeneration of frontal cortex, especially prefrontal areas, have been related to impairment of executive functions. In our study, patients with dementia were classified as a moderate level of severity, possibly corresponding to Braak stages 4 or 5 which involve midbrain nuclei as well as neocortical regions. However, this classification is only a supposition since definitive identification of tissue lesions requires histological examination.

Similarly to previous studies, we used the CAMCOG test to assess overall cognitive performance and simultaneously used the Stroop test to cover specific cognitive domains, such as executive function subscales,^28,29^ particularly the attention process.^17,24,30^ As expected, the patients with dementia showed worse cognitive performance on the CAMCOG and Stroop test – ‘time’ category and were therefore cognitively slower. However, there was no difference between the groups with and without dementia on the ‘word errors’ category of the Stroop test, which measures attention and abstraction. In addition to the relatively small number of subjects, the wide range of patients’ scores observed should be noted, with a consequent high standard deviation. This combination of factors may partially explain this surprising finding. Furthermore, all patients with dementia had poor performance in evaluation of frontal functions on the CAMCOG, mainly in abstract thinking and the attention process.

Patients with late onset of PD tend to have an accelerated evolution of the disease, thus cognitive and functional decline may manifest more rapidly and intensely. This phenomenon results from a swift neurodegenerative process in the brain.^1^ In our study, there was no difference related to age at onset of PD between the groups; however, the patients with dementia had a longer period of illness, in contrast to the findings observed by the above-mentioned authors mentioned.^1,23-25,30^

Regarding clinical manifestations, patients with predominant rigidity, postural instability and gait difficulty usually have faster disease progression and tend to be more cognitively impaired; on the other hand, those with tremor predominance tend to have slower illness progression and a more preserved cognition.^1^ Several earlier studies have showed association between cognitive decline and rigidity or instability.^1,5,14,31,32^ Moreover, Jankovic & Kapadai^1^ found that the tremor subtype of PD was associated with preserved mental condition, earlier age at onset and slower progression of the disease in comparison with postural instability and gait difficulty, which were associated with cognitive impairment, more severe bradykinesia, and a more rapid progression of PD. In addition to postural instability and gait disturbances, Emre et al.^14^ and Burn et al.^32^ found an association between rigidity and cognitive impairment in patients with PD. Evolution of dementia in patients with more severe motor symptoms such as postural instability and gait difficulty have been associated to rate of neurodegeneration and cell loss in several neurochemical systems, in addition to more rapid cognitive decline.^32^

Although most authors have verified associations between the clinical features outlined and cognitive impairment, others have found a link between the presence of tremors as a predominant condition and cognitive impairment. According to Vingerhoets et al.,^33^ patients with tremor at disease onset are more likely to suffer cognitive impairment in the more advances stages of PD compared to patients with akinesia or rigidity at onset. Although the reasons for these contradictory findings remain unclear, according to these authors, tremor is a more resistant condition to dopaminergic treatment than other motor symptoms, and probably pathological involvement of non-dopaminergic areas of the brain are responsible. Furthermore, tremor at onset of PD may be considered as a marker for widespread brain disease, especially for injury of non-dopaminergic pathways that contribute to an increased risk for cognitive impairment, in particular for disturbances in memory and attention.^33^ These authors consider that the tremor at onset symptom and tremor as a predominant symptom of PD are distinct conditions that occur at different points in the disease course, and this fact could partially explain the contradictory results. In our study, there was a relatively homogeneous distribution between the groups in terms of onset of PD, symptom type (tremor or rigidity) and beginning of the disease in hemibody. The small size of our sample, however, prevented further analysis in this regard. It should be noted that similar results were obtained by Papapetropoulos et al.,^34^ who studied PD patients with and without dementia and did not find significant differences in cognition related to motor features such as tremor, bradykinesia and rigidity, freezing or falls.

In summary, compared with cognitively preserved PD patients, those with dementia had a longer period of PD, and were found to be at a more advanced clinical stage and to present greater functional impairment. Patients without dementia had more decline in activities of daily living compared to patients with dementia. The data suggest that dementia is an aggravating condition for functional impairment. Further studies are needed in order to ascertain to what extent impairment in specific cognitive domains, particularly executive functions, contribute to the overall functional decline in PD patients with dementia.

## References

[1] Jankovic J, Kapadia AS (2001). Functional decline in Parkinson disease. Arch Neurol.

[2] Potagas C, Papageorgiou SG (2006). Phenomenology and management of cognitive and behavioral
disorders in Parkinson's disease: rise and logic of dementia in Parkinson's
disease. An Gen Psychiatry.

[3] Lauderbach EC (2004). The neuropsychiatry of Parkinson's disease and related
disorders. Psychiatr Clin North Am.

[4] Sabbagh MN, Silverberg N, Bircea S (2005). Is the functional decline of Parkinson's disease similar to the
functional decline of Alzheimer's disease?. Parkinsonism Relat Disord.

[5] Giladi N, Treves TA, Paleacu D (2000). Risk factors for dementia, depression and psychosis in
long-standing Parkinson's disease. J Neurol Transm.

[6] Fahn S, Elton R, Fahn S, Marsden CD, Calne DB, Goldstein M, Members of the UPDRS, Development Committee (1987). The unified Parkinson's disease rating scale. Recent developments in Parkinson's disease.

[7] Schwab RS, England AC, Gillingham FJ, Donaldson ML (1969). Projection technique for evaluating surgery in Parkinson's
disease. Third Symposium on Parkinson's Disease.

[8] Weintraub D, Moberg PJ, Duda JE, Katz IR, Stern MB (2004). Effect of psychiatric and other non-motor symptoms on disability
in Parkinson's disease. J Am Geriatr Soc.

[9] Hughes AJ, Daniel SE, Kilford L, Lees AJ (1992). Accuracy of clinical diagnosis of idiopathic Parkinson's disease:
a clinico-pathological study of 100 cases. J Neurol Neurosurg Psychiatry.

[10] Roth M, Tym E, Mountjoy CQ (1986). CAMDEX - A standardized instrument for the diagnosis of mental
disorder in the elderly with special reference to the early detection of
dementia. Brit J Psychiatry.

[11] Bottino CMC, Stoppe Jr A, Scalco AZ (2001). Validade e confiabilidade da versão brasileira do
CAMDEX. Arq Neuropsiquiatr.

[12] Gazzainiga MS, Ivry RB, Mangun GR (1998). Cognitive Neuroscience: The Biology of the Mind.

[13] Dornelles Cláudia, American Psychiatric Association (2003). Manual Diagnóstico e Estatístico dos Transtornos
Mentais.

[14] Emre M, Aarsland D, Brown R (2007). Clinical diagnostic criteria for dementia associated with
Parkinson's disease. Mov Disord.

[15] Hoehn MM, Yarh MD (1967). Parkinsonism: Onset, progression and mortality. Neurology.

[16] Marras C, McDermott MP, Rochon PA, Tanner CM, Naglie G, Lang AE (2007). Predictors of deterioration in health-related quality of life in
Parkinson's disease: Results from the DATATOP trial. Mov Disord.

[17] Janvin CC, Aarsland D, Larsen JP (2005). Cognitive predictors of dementia in Parkinson's disease: a
community-based, 4-year longitudinal study. J Geriatr Psychiatr Neurol.

[18] Martinez-Martin P, Prieto L, Forjaz MJ (2006). Longitudinal metric properties of disability rating scales for
Parkinson's disease. Value Health.

[19] Forjaz MJ, Martinez-Martin P (2006). Metric attributes of the Unified Parkinson's Disease Rating Scale
3.0 Battery: Part II, construct and content validity. Mov Disord.

[20] Carod-Artal FJ, Vargas AP, Martinez-Martin P (2007). Determinants of quality of life in Brazilian with Parkinson's
disease. Mov Disord.

[21] Calabresi P, Picconi B, Parnetti L, Di Filippo M (2006). A convergent model for cognitive dysfunction in Parkinson's
disease: the critical dopamine-acetylcholine synaptic
balance. Lancet Neurol.

[22] Galvin JE (2006). Cognitive change in Parkinson disease. Alzheimer Dis Assoc Disord.

[23] Braak H, Rub U, Del Tredici K (2006). Cognitive decline correlates with neuropathological stage in
Parkinson's disease. J Neurol Sci.

[24] Bohnen NI, Kaufer DI, Hendrickson R (2006). Cognitive correlates of cortical cholinergic denervation in
Parkinson's disease and parkinsonian dementia. J Neurol.

[25] Jellinger KA (2006). The morphological basis of mental dysfunction in Parkinson's
disease. J Neurol Sci.

[26] Zgaljardic DJ, Foldi NS, Borod JC (2004). Cognitive and behavioral dysfunction in Parkinson's disease:
neurochemical and clinicopathological contributions. J Neural Transm.

[27] Braak H, Del Tredici K, Rub U, de Vos RAI, Jansen Steur ENH, Braak E (2003). Staging of brain pathology related to sporadic Parkinson's
disease. Neurobiol Aging.

[28] Hobson P, Meara J (1999). The detection of dementia and cognitive impairment in a community
population of elderly people with Parkinson's disease by use of the CAMCOG
neuropsychological test. Age Aging.

[29] Athey RJ, Walker RW (2006). Demonstration of cognitive decline in Parkinson's disease using
the Cambridge Cognitive Assessment (Revised) (CAMCOG-R). Int J Geriatr Psychiatry.

[30] Dujardin K, Defebvre L, Krystkowiak P, Degreef JF, Destee A (2003). Executive function differences in multiple system atrophy and
Parkinson's disease. Parkinsonism Relat Disord.

[31] Hughes TA, Ross HF, Musa S (2000). A 10-year study of the incidence of and factors predicting
dementia in Parkinson's disease. Neurology.

[32] Burn DJ, Rowan EN, Allan LM, Molloy S, O'Brien JT, McKeith IG (2006). Motor subtype and cognitive decline in Parkinson's disease,
Parkinson's disease with dementia, and dementia with Lewy
bodies. J Neurol Neurosurg Psychiatry.

[33] Vingerhoets G, Verleden S, Santens P, Miaton M, De Reuck J (2003). Predictors of cognitive impairment in advanced Parkinson's
disease. J Neurol Neurosurg Psychiatry.

[34] Papapetropoulos S, Gonzalez J, Lieberman A, Villar JM, Mash DC (2005). Dementia in Parkinson's disease: a post mortem study in a
population of brain donors. Int J Geriatr Psychiatry.

